# Advanced interstitial chemotherapy combined with targeted treatment of malignant glioma in rats by using drug-loaded nanofibrous membranes

**DOI:** 10.18632/oncotarget.10989

**Published:** 2016-08-01

**Authors:** Yuan-Yun Tseng, Chen-Hsing Su, Shun-Tai Yang, Yin-Chen Huang, Wei-Hwa Lee, Yi-Chuan Wang, Shou-Cheng Liu, Shih-Jung Liu

**Affiliations:** ^1^ Division of Neurosurgery, Department of Surgery, Shuang Ho Hospital, Taipei Medical University, Taipei, Taiwan; ^2^ Department of Surgery, School of Medicine, College of Medicine, Taipei Medical University, Taipei, Taiwan; ^3^ Department of Neurosurgery, Chung Shan Medical University Hospital, Taichung, Taiwan; ^4^ Department of Neurosurgery, Chang Gung Memorial Hospital-Chiayi, Chang Gung University College of Medicine, Tao-Yuan, Taiwan; ^5^ Department of Pathology, Shuang Ho Hospital, Taipei Medical University, Taipei, Taiwan; ^6^ Department of Mechanical Engineering, Chang Gung University, Tao-Yuan, Taiwan; ^7^ Department of Orthopedics, Chang Gung Memorial Hospital, Tao-Yuan, Taiwan

**Keywords:** glioblastoma multiforme (GBM), chemotherapy, targeted therapy, nanofiber, antiangiogenesis

## Abstract

Glioblastoma multiforme (GBM), the most prevalent and malignant form of a primary brain tumour, is resistant to chemotherapy. In this study, we concurrently loaded three chemotherapeutic agents [bis-chloroethylnitrosourea, irinotecan, and cisplatin; BIC] into 50:50 poly[(d,l)-lactide-co-glycolide] (PLGA) nanofibres and an antiangiogenic agent (combretastatin) into 75:25 PLGA nanofibres [BIC and combretastatin (BICC)/PLGA]. The BICC/PLGA nanofibrous membranes were surgically implanted onto the brain surfaces of healthy rats for conducting pharmacodynamic studies and onto C6 glioma-bearing rats for estimating the therapeutic efficacy.

The chemotherapeutic agents were rapidly released from the 50:50 PLGA nanofibres after implantation, followed by the release of combretastatin (approximately 2 weeks later) from the 75:25 PLGA nanofibres. All drug concentrations remained higher in brain tissues than in the blood for more than 8 weeks. The experimental results, including attenuated malignancy, retarded tumour growth, and prolonged survival in tumour-bearing rats, demonstrated the efficacy of the BICC/PLGA nanofibrous membranes. Furthermore, the efficacy of BIC/PLGA and BICC/PLGA nanofibrous membranes was compared. The BICC/PLGA nanofibrous membranes more efficiently retarded the tumour growth and attenuated the malignancy of C6 glioma-bearing rats. Moreover, the addition of combretastatin did not significantly change the drug release behaviour of the BIC/PLGA nanofibrous membranes. The present advanced and novel interstitial chemotherapy and targeted treatment provide a potential strategy and regimen for treating GBM.

## INTRODUCTION

Glioblastoma multiforme (GBM) has been the most prevalent and malignant type of cancer in the central nervous system (CNS). GBM has a median survival rate of around 12–15 months when standard treatment is administered [1, 2]. However, the median survival rate of patients with GBM has not significantly changed, despite more than two decades of researches on the development of various agents and delivery systems [3–5]. The tumour location is associated with the poor penetration of drugs through the blood–brain barrier (BBB) and also within the tumour. Moreover, established resistance to chemotherapy and radiation therapy; the highly infiltrative nature of tumour cells; genetic, morphological, and molecular heterogeneity; and an extremely developed but inadequately functioning neovasculature contribute to a poor prognosis in patients with GBM [4, 6, 7].

GBM develops a unique neovasculature which is highly permeable to macromolecules and small particles. The disruption of the BBB is a regional incident occurring in the tumour core, whereas in its growing margins the BBB remains intact. Therefore, typically, the efficacy of systemic drug therapy cannot be completely realised since most anti-carcinoma agents fail to cross the BBB and thus do not reach therapeutic concentrations in the tumour [8–10].

Interstitial chemotherapy is able to deliver high strengths of chemotherapeutic agents directly to the brain tumour with minimum systemic toxicities [11]. Carmustine or 1,3-bis(2-chloroethyl)-1-nitrosourea (BCNU) wafers (Gliadel; Guilford Pharmaceuticals, Baltimore, MD, USA) show an increasingly popular approach for delivering chemotherapy, theoretically supplying high concentrations of chemotherapeutic doses locally while minimising systemic adverse effects [12, 13]. These biodegradable, BCNU-impregnated polymers are implanted in the tumour bed during resection, thus providing a controlled release of local chemotherapy. However, patients who received the Gliadel wafer treatment survived only about 2 months longer than those who did not receive the treatment [14, 15]. Interstitial chemotherapy using biodegradable polymers that are directly delivered to the brain appears to be a safe and efficacious therapy for recurrent or newly diagnosed high-level gliomas, but the therapeutic effect requires improvement for yielding greater survival benefits.

Cisplatin is a platinum compound consisted of two chloride atoms and two amine groups. Once it is introduced into the body, water molecules will displace the chloride atoms; leading to the crosslinking of the hydrated complex crosslinks with DNA strands and triggering programmed cell death. Cisplatin reduces the *in vitro* arginine:glycine amidinotransferase (AGAT) activity and is considered as an active agent, in addition to nitrosoureas, for treating glioma [16]. Furthermore, irinotecan, a camptothecin derivative that restrains topoisomerase I, is an indispensable enzyme necessary for the relaxation of supercoiled DNA, which causes topological variations that promote RNA transcription and DNA replication [17]. Irinotecan is approved for treating metastatic colorectal cancer and is a recommended drug for treating the recurrent and intractable forms of this disease that has evolved despite medical treatment with fluorouracil [18]. Moreover, combretastatin A4 (CA4) phosphate (CA4P), a tubulin-binding vascular disrupting agent, targets the existing vasculature of tumours, causing a rapid vascular shutdown, resulting in cell death and central necrosis [19, 20]. Combretastatin is undergoing phase II trials for use in treating ovarian, lung, and anaplastic thyroid cancer [19, 21].

In this work, the authors concurrently loaded the BCNU, irinotecan, and cisplatin (BIC) into 50:50 poly[(d,l)-lactide-co-glycolide] (PLGA) nanofibres and combretastatin into 75:25 PLGA nanofibres. The *in vivo* drug concentration was examined. Thereafter, the anticancer agents embedded nanofibrous membranes were implanted onto the brain surfaces of glioma-bearing rats for administering combined (drug cocktail) chemotherapy and sequential targeted therapy. The tumour growth rate, rat survival rate, and pathological data were evaluated for determining the therapeutic efficacy of the drug-loaded nanofibres and their potential in treating GBM.

## RESULTS

Drug-loaded nanofibrous membranes were successfully prepared using suitable processing conditions. Scanning electron microscopy (SEM) photos of the nanofibres, under 5000× magnification, suggested that the diameters of the electrospun drug-loaded PLGA nanofibres ranged between 375 and 1200 nm with a high porosity.

### *In vivo* release behaviours of chemotherapeutic pharmaceuticals from nanofibres

**Figure 1:**
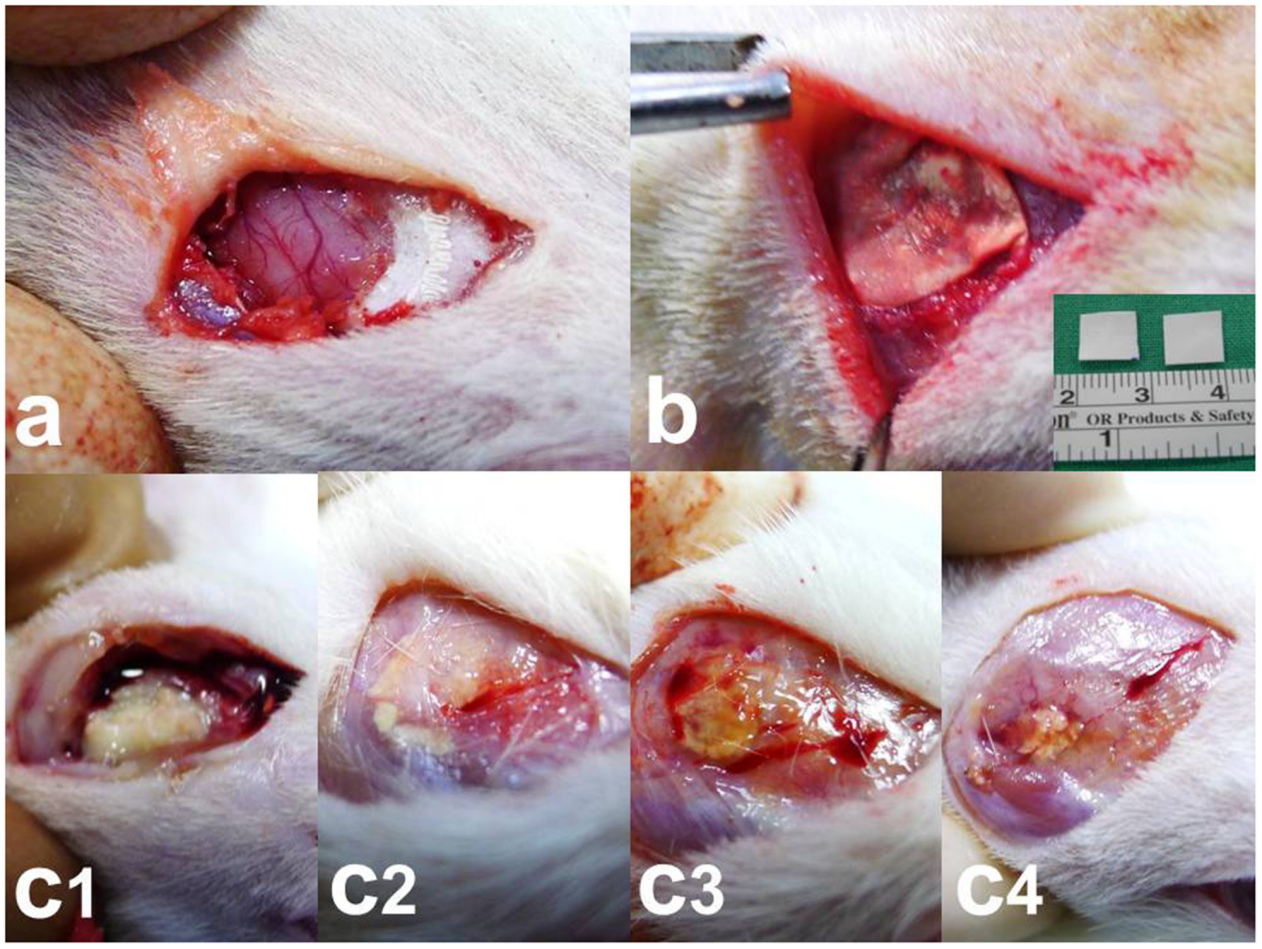
Surgical procedure and nanofibrous membrane degradation **a.** Craniectomy (approximately 1 × 1 cm) was performed using an electric burr. **b.** Nanofibrous membranes (0.8 × 0.8 cm) were implanted onto the brain surface of rats. **c1–4.** The nanofibrous membranes gradually degraded. c1. 2 weeks, c2. 4 weeks, c3. 6 weeks, and c4. 8 weeks. A low number of residual nanofibrous membranes was observed at the end of the study (8 weeks).

**Figure 2:**
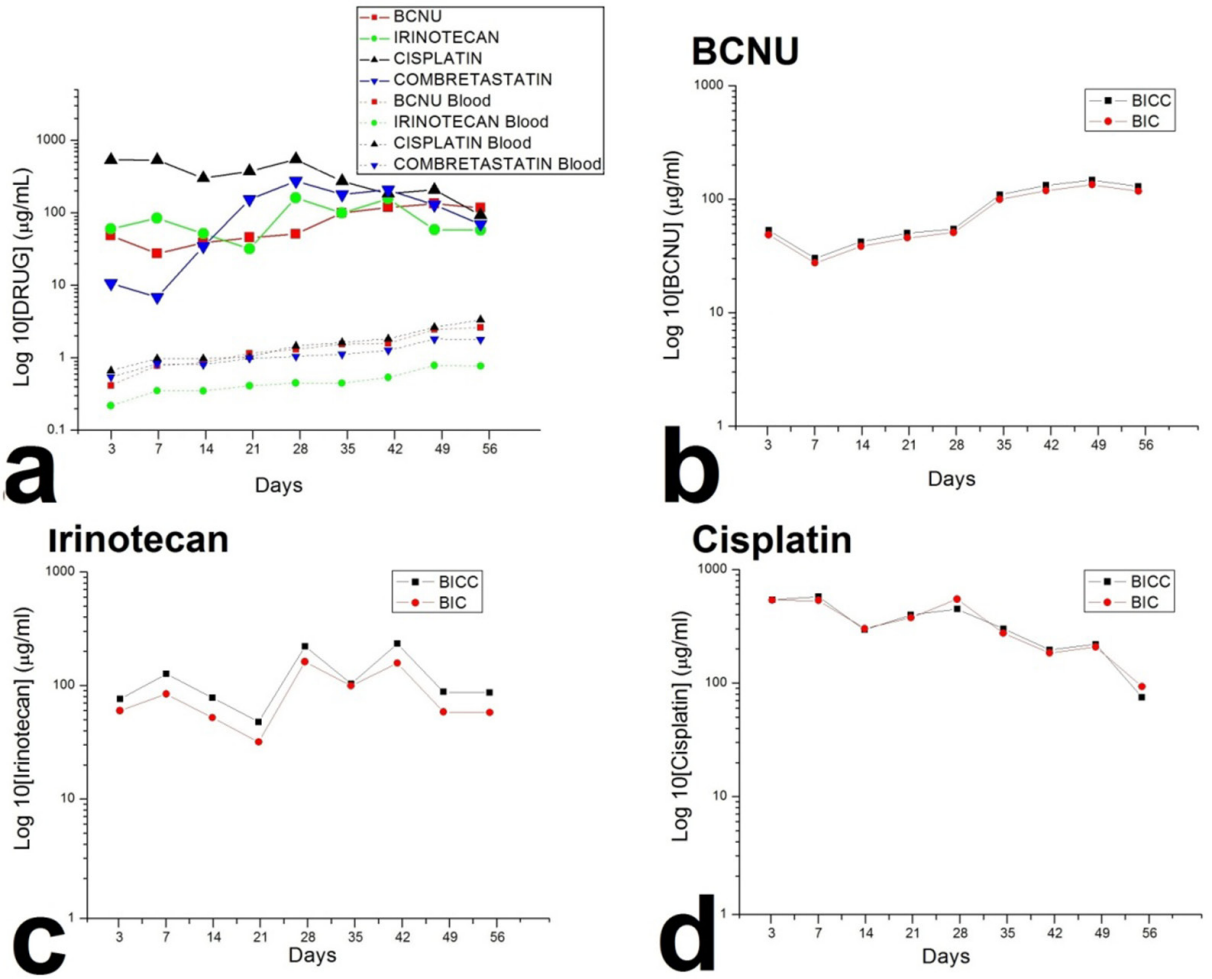
Drug concentrations in the brain tissues and the blood **a.** The chemotherapeutic agents were rapidly released from the 50:50 PLGA nanofibrous membranes, and the antiangiogenic agent was released from the 75:25 PLGA nanofibrous membranes approximately 2 weeks later. The drug concentration in the brain was higher than that in the blood for more than 8 weeks. **b–d.** The release behaviour of all the chemotherapeutic agents from the BICC/PLGA nanofibrous membranes was comparable to that from the BIC/PLGA nanofibrous membranes, with no statistical difference.

After the rats that succumbed during the perioperative period (due to anaesthesia overdose or massive blood loss) and those with brain injury, including brain, wounds, or systemic infection were excluded, at least five rats at each time point were enrolled for analysing BIC and combretastatin (BICC) concentrations. *In vivo* concentrations were characterised for 8 weeks through high-performance liquid chromatography. The BICC/PLGA nanofibrous membranes progressively degraded, yielding small residual PLGA nanofibres at the end of the study (Figure 1 c1–c4). Furthermore, the *in vivo* release characteristics of BICC from the anticancer agents embedded nanofibers are presented in Figure 2. The chemotherapeutic agents were rapidly released from the BICC/PLGA nanofibrous membranes. On Day 3, the cisplatin concentration was the highest (660.47 ± 406.71 μg/mL), and the drug concentrations in the blood were the lowest (0.67 ± 0.13 μg/mL); the brain–blood drug concentration ratio was 985.78. For all the chemotherapeutic agents, the brain–blood drug concentration ratios reached their maximum values on Day 3 and were 115.76 and 272.59 times for BCNU and irinotecan, respectively. The drug concentrations in brain tissues remained high (approximately 100 μg/mL) and exceeded those in the blood during the entire study (8 weeks). Furthermore, the drug concentrations in the blood slowly increased with the degrading PLGA nanofibres and reached the highest concentration on Day 56. All brain–blood drug concentration ratios were at their minimum values on Day 56 and were 44.88, 77.03, and 20.89 times for BCNU, irinotecan, and cisplatin, respectively. However, the concentrations of BCNU, irinotecan, and cisplatin were considerably higher in the cerebral cavity than in the plasma (systemic). Drug concentrations differences between the brain and blood reached significance at every time point (P < 0.01). Furthermore, the release behaviour of all the chemotherapeutic agents from the BICC/PLGA nanofibrous membranes was comparable with that from the BIC/PLGA nanofibrous membranes, with no statistical difference (Figure 2). In the first 2 weeks, the combretastatin concentration remained low (below 10 μg/mL), thereafter progressively increasing to more than 100 μg/mL in the third week (152.82 ± 73.13 μg/mL) and reaching the maximum concentration in the fourth week (274.68 ± 113.17 μg/mL), with the highest brain–blood drug concentration ratio (263.46). The high brain–blood drug concentration ratio lasted until the end of the study (39.12 in the eighth week).

### Survival rate

**Figure 3:**
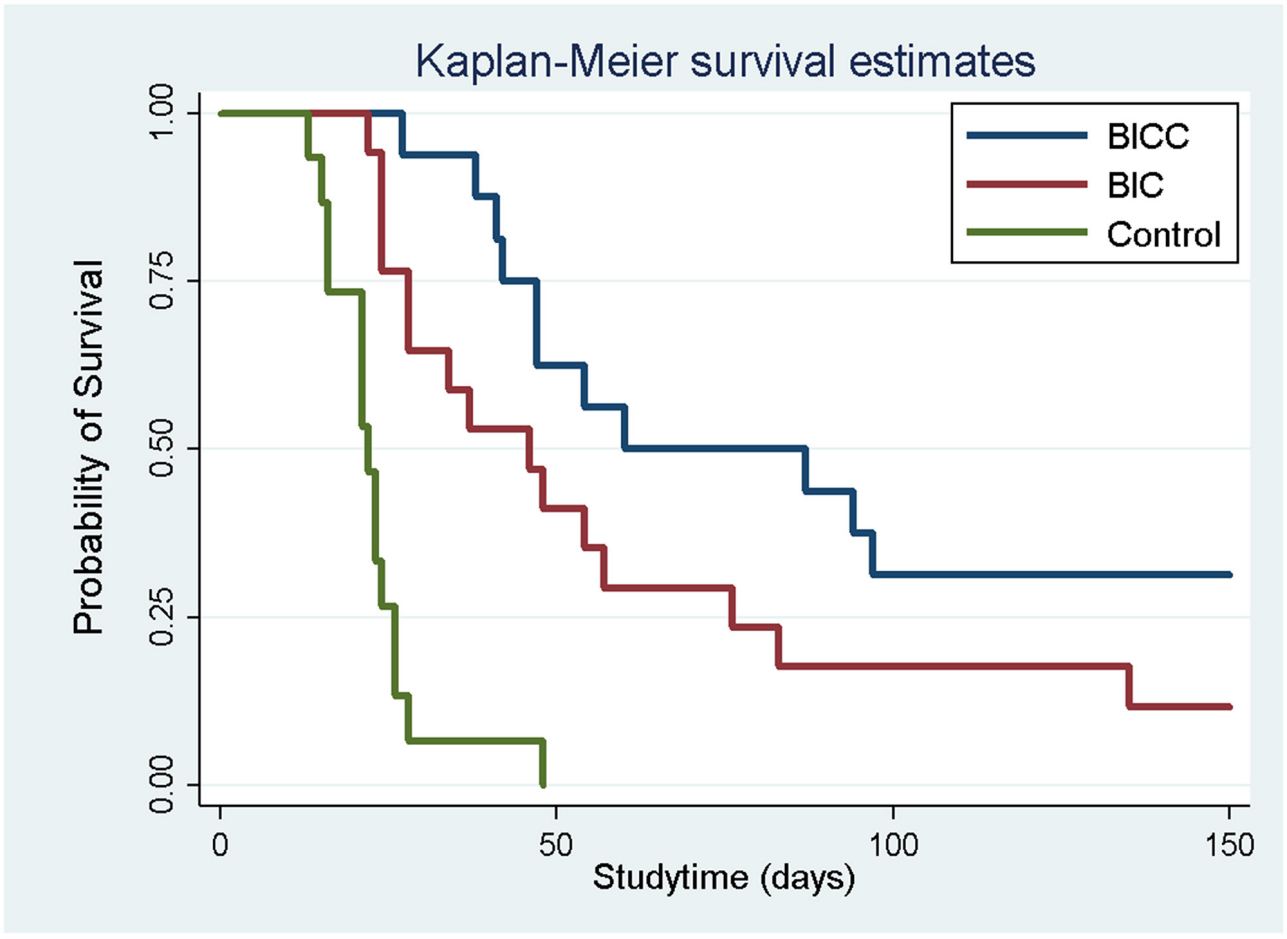
Survival analysis The survival rates for all the groups were analysed using the Kaplan–Meier method. No difference was observed between the BICC and BIC groups (P = 0.08). The overall survival rate was significantly higher in the drug-loaded groups (BICC versus control group, P < 0.001; BIC versus control group, P < 0.001).

The rats that died during the perioperative period, developed wound infection, or failed to develop glioma were excluded. A total of 56 glioma rat models were successfully created, which was confirmed through brain magnetic resonance imaging (MRI). Excluding the eight tumour-bearing rats that were sacrificed for pathological examination, 15, 17, and 16 rats were included in the control, BIC, and BICC groups, respectively. In the control group, 14 rats died within 4 weeks of tumour implantation; only one rat survived for more than 4 weeks and died on Day 56. The median survival time was 22.87 ± 8.21 days in the control group, whereas the median survival times were 60.00 ± 44.43 days and 86.50 ± 48.41 days in the BIC and BICC groups, respectively. Moreover, the survival period was significantly shorter in the control group than in the BIC (P = 0.026) and BICC groups (P < 0.001). Furthermore, the Kaplan–Meier survival curves in Figure 3 suggested that the overall survival rate was higher in the BIC and BICC groups than in the control group (P < 0.001). Nevertheless, the difference did not show statistical significance (P = 0.077).

### MRI examination

Approximately 10–12 days after C6 glioma cell implantation into the rat brains, T1- and T2-weighted images were taken for confirming the successful creation of the glioma models. A series of brain MRI inspections were completed before the membrane being implanted and at 0, 2, 4, 6, 8, 10, 14, 18, and 22 weeks after the implantation. A high percentage (43.75%) of the rats in the BICC group exhibited complete responses, and 31.25% exhibited partial responses, whereas 25% of the rats exhibited no response. Furthermore, in the BIC group, approximately half of the rats (47.06%) exhibited partial responses, a similar percentage (46.67%) of them exhibited no response, and only two rats (11.76%) displayed complete responses. Relatively, no animals in the control group showed a complete response; only one rat displayed a partial response, and most rats (93.33%) rapidly died (Table 1).

**Table 1:** Responses of the rats to various treatments

	Rats by treatment group		
	BICC N = 16)	BIC (N = 17)	Control (N = 15)
**Response data**	No. (%)	No. (%)	No. (%)
**Complete**	7 (43.75)	2 (11.76)	0 (0)
**Partial**	5 (31.25)	8 (47.06)	1 (6.67)
**No**	4 (25.00)	7 (46.67)	14 (93.33)

**Figure 4:**
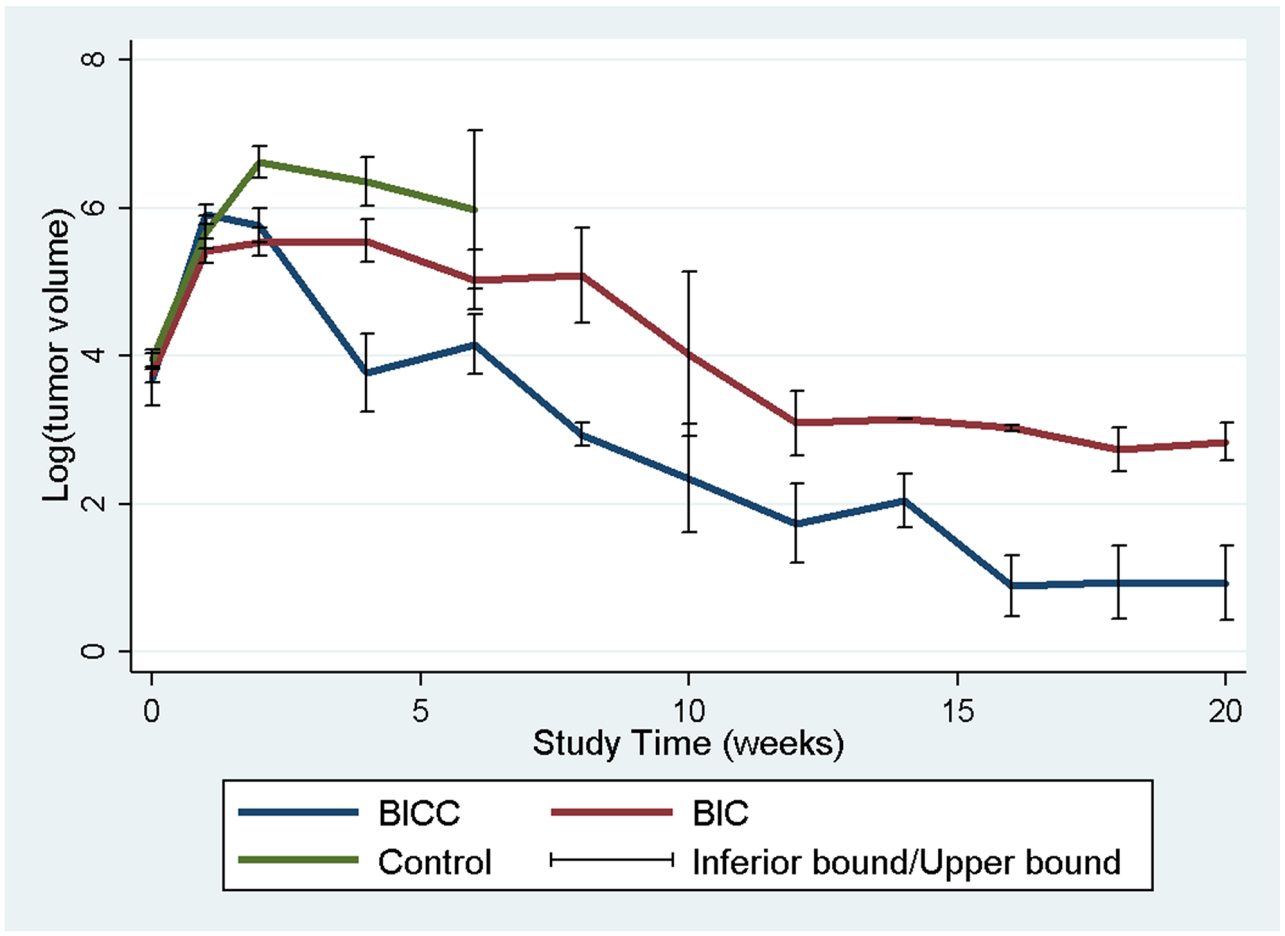
Tumour volumes A repeated measures mixed model was used for evaluating the tumour volume change in the three groups. The tumour volumes in the control group rapidly increased and were significantly higher than those in the BIC and BICC groups (control versus BIC group, P = 0.004; control versus BICC group, P < 0.001). The mean tumour volumes were significantly lower in the BICC group than in the BIC group (P = 0.045).

The tumour volumes were reconstructed and analysed employing the open-sourced, Food and Drug Administration (FDA)-approved Digital Imaging and Communication in Medicine software OsiriX. The mean tumour volumes before the membrane implantation (approximately 10–12 days after tumour cell incubation) were 60.36± 38.69 × 10^−3^ mL, 46.32 ± 19.32 × 10^−3^ mL, and 59.12 ± 321.19 × 10^−3^ mL in the control, BIC, and BICC groups, respectively. The mean tumour volumes were lower in the BIC group; however, the difference was not statistically significant. After the implantation, the tumour volumes rapidly increased in the control group, with tumour volumes of 418.13 ± 396.10 × 10^−3^ mL, 866.00 ± 498.52 × 10^−3^ mL, 719.07 ± 430.94 × 10^−3^ mL, and 647.07 ± 723.26 × 10^−3^ mL at 1, 2, 4, and 6 weeks, respectively. The tumour volumes also rapidly increased in the first week in the BICC group, and the estimated tumour volume was 401.99 ± 162.61 × 10^−3^ mL. Furthermore, the tumour volumes slightly increased and reached the maximum value (427.98 ± 392.92 × 10^−3^ mL) at 2 weeks; thereafter, the tumour volumes clearly decreased, with a mean volume of 140.59 ± 212.44 × 10^−3^ mL, 119.55 ± 149.02 × 10^−3^ mL, 20.44 ± 9.40 × 10^−3^ mL, 6.68 ± 6.34 × 10^−3^ mL, and 2.46 ± 2.97 × 10^−3^ mL at 4, 6, 8, 12, and 16 weeks, respectively. The tumour volumes increased more slowly in the BIC group, with a volume of 272.05 ± 207.05 × 10^−3^ mL in the first week, which was less than the volume in the control and BICC groups (P = 0.429, statistically nonsignificant). Moreover, the tumour volumes slowly increased and reached the maximum value (401.44 ± 460.62 × 10^−3^ mL) in the fourth week. Furthermore, the repeated measures mixed model analysis of tumour volumes in the control, BIC, and BICC groups is presented in Figure 4. The tumour volumes rapidly increased in the control group, whereas the volumes gradually increased in the first 4 weeks and decreased thereafter in the BIC group. In the BICC group, the tumour volumes increased and reached the maximum value at 2 weeks and then decreased more than that in the BIC group. The difference among the three groups was statistically significant (BIC versus BICC group, P = 0.045; BICC versus control group, P < 0.001; and BIC versus control group, P = 0.004).

**Figure 5:**
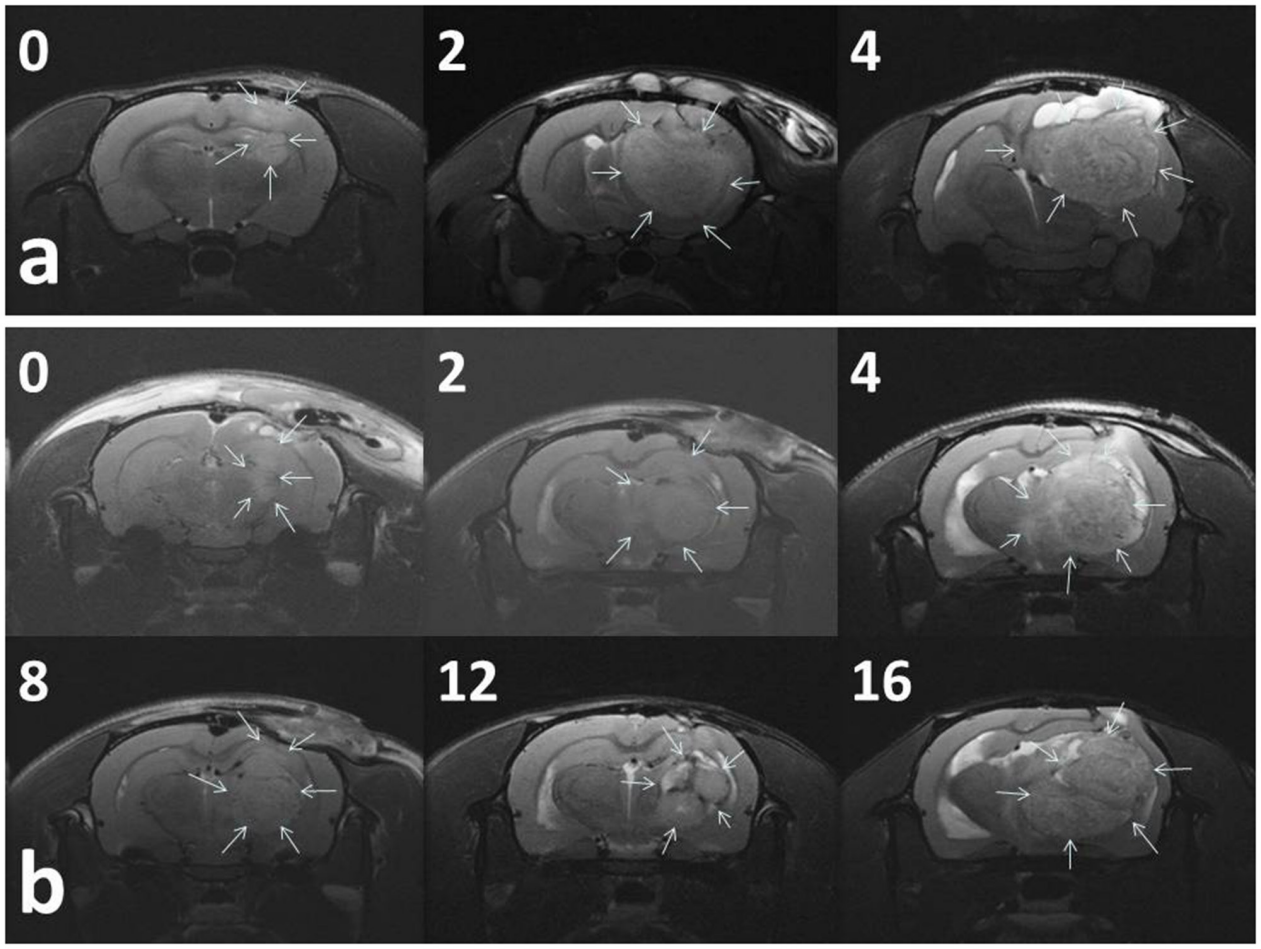
Serial MRI images in the control group A. and BIC group B The number in the upper right corner of each image indicates the number of weeks after the implantation of nanofibrous membranes. **a.** The tumour volumes of most (93.33%) of the rats in the control group rapidly increased and resulted in a severe mass effect and death. **b.** The rats in the BIC group exhibited a partial response to BIC/PLGA nanofibrous membrane treatment, and the tumour volumes temporarily decreased between 4 and 12 weeks, followed by a rapid increase in the volume.

**Figure 6:**
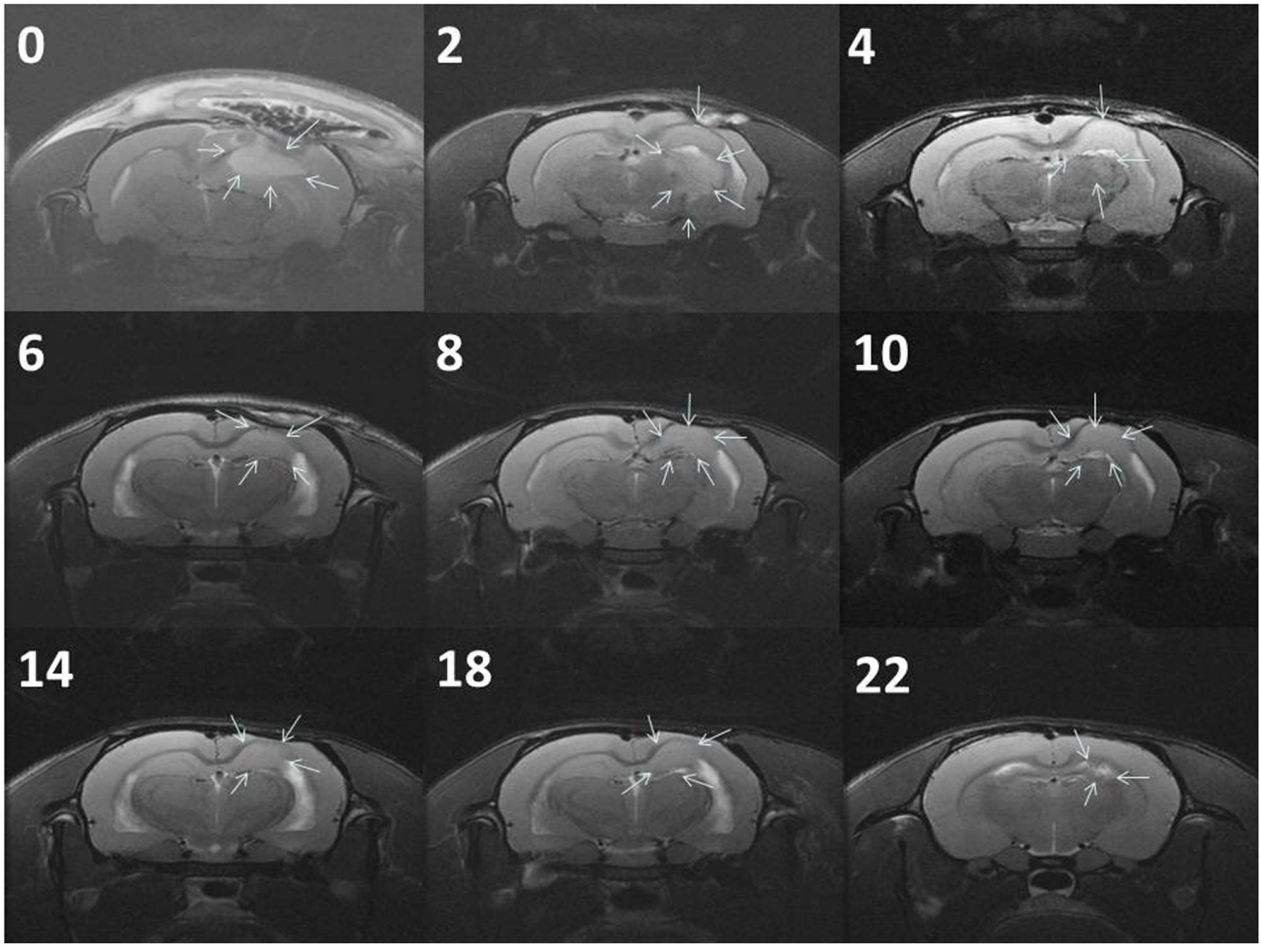
Serial MRI images of the BICC group The rats exhibited a complete response to BICC/PLGA nanofibrous membrane treatment. The number in the upper right corner of each image indicates the number of weeks after the implantation of BICC/PLGA nanofibrous membranes. The tumour volumes clearly decreased with time, and no tumour regrowth was observed.

Figure 5a presents the serial MRI scans of the animals in the control group. The implanted tumour rapidly grew and resulted in an intense mass effect (midline shift and brain stem compression), eventually causing the rats to die. Figure 5b illustrates the serial brain MRI scans of the animals in the BIC group, in which the created tumour grew more slowly than in the animals in the control group. The tumour volumes decreased between Weeks 4 and 8 and thereafter progressively regrew and caused the death of the rats in Week 23. Figure 6 presents the serial brain MRI scans of the rats in the BIC group. The initial tumour volume (62.73 × 10^−3^ mL) was similar to the mean tumour volume in the control group (60.36 ± 38.69 × 10^−3^ mL). The tumour slowly grew and reached the maximum value in Week 4 and thereafter decreased steadily and nearly disappeared at the end of the study.

### Pathology

Table 2 presents the haematoxylin and eosin (H & E) staining and immunohistochemical (glial fibrillary acidic protein [GFAP] and Ki-67) results of the three groups. H & E staining revealed that the central necrotic and tumour areas rapidly and markedly increased in the control group (a and b). The central necrotic area expanded more slowly in the BIC group, and some calcification was observed (c and d). The central necrotic and tumour infiltration areas decreased in the BICC group (e and f). Moreover, GFAP expression markedly decreased in the control group (g and h). The GFAP expression was suppressed in the BIC (c and d) and BICC (e and f) groups; the suppression was higher in the BIC group. In both the BIC and BICC groups, the GFAP expression progressively increased. The Ki-67 labelling index was 22.84% ± 7.93% and 45.98% ± 11.57% in Weeks 4–6 and 8–12, the Ki-67 index was high and markedly increased in the control group. By contrast, this index was 15.98% ± 7.31% in Weeks 4–6 and decreased to 11.41% ± 4.13% in Weeks 8–12 in the BIC group. This index was much lower in the BICC group than that in the control and BIC groups (7.29% ± 1.35% and 0.79% ± 0.55% in Weeks 4–8 and 10–14, respectively).

**Table 2:** Results of histological examination

Stain	Gr.	4-6 weeks	8-12 weeks
H&E	Control	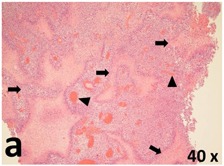	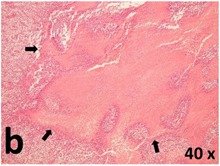
	BIC	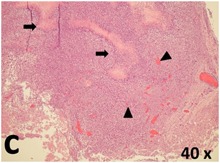	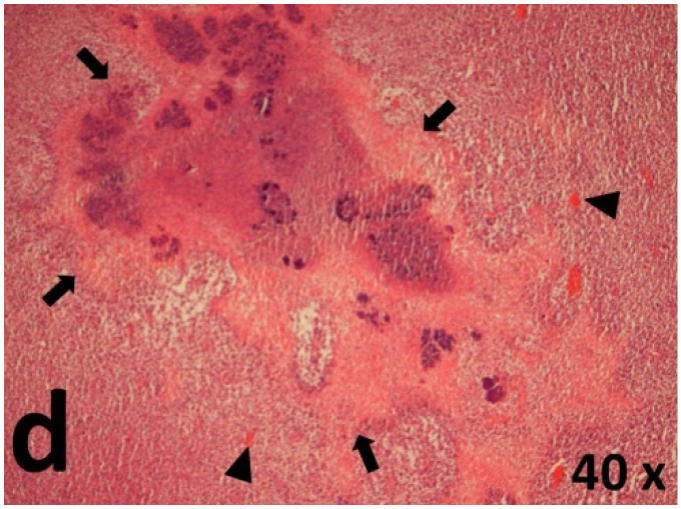
	BICC	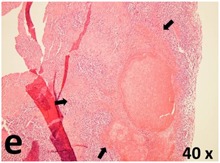	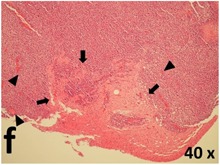
GFAP	Control	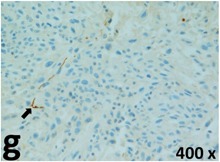	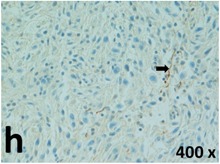
	BIC	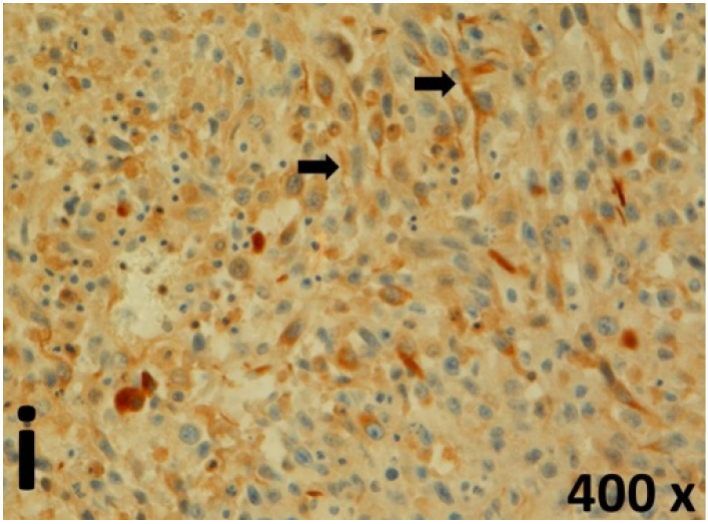	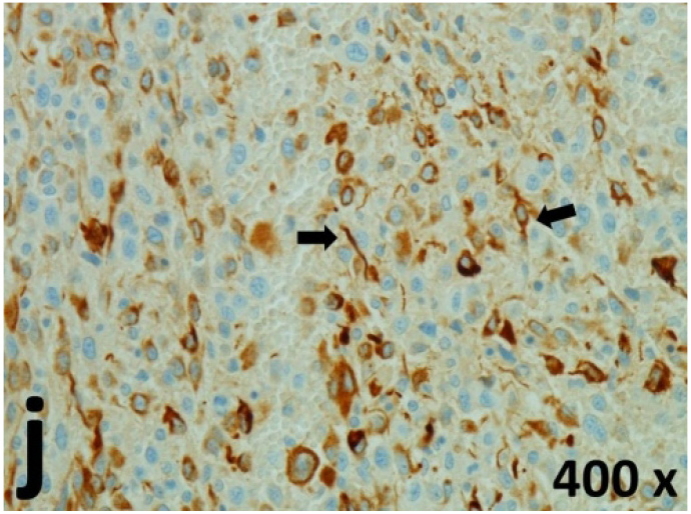
	BICC	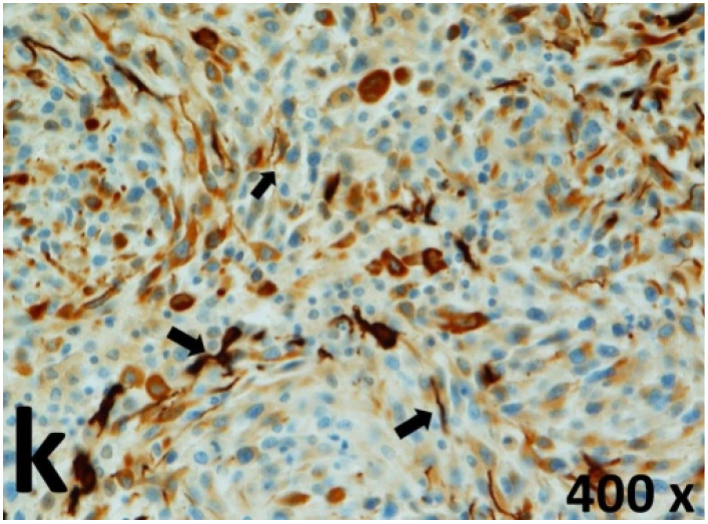	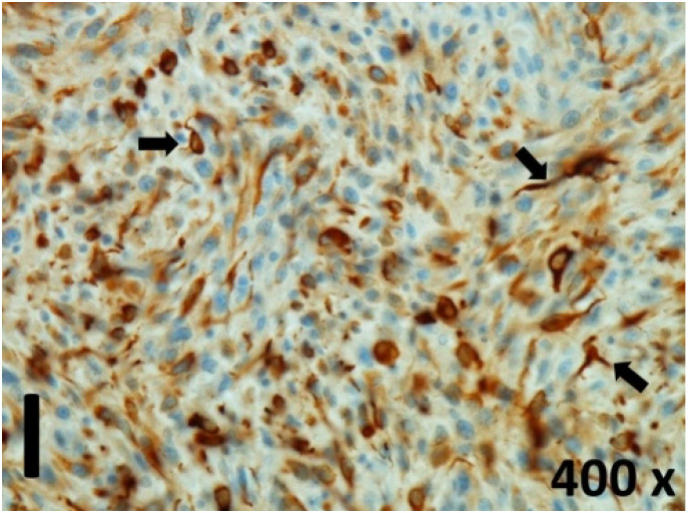
Ki-67	Control	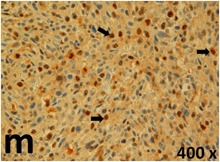	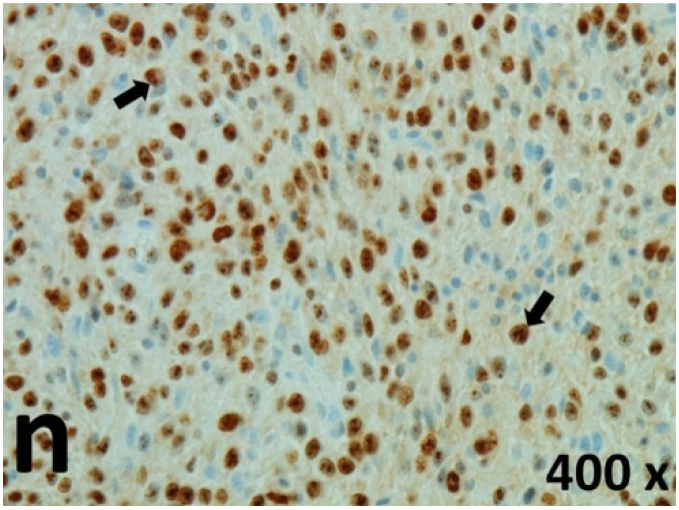
	BIC	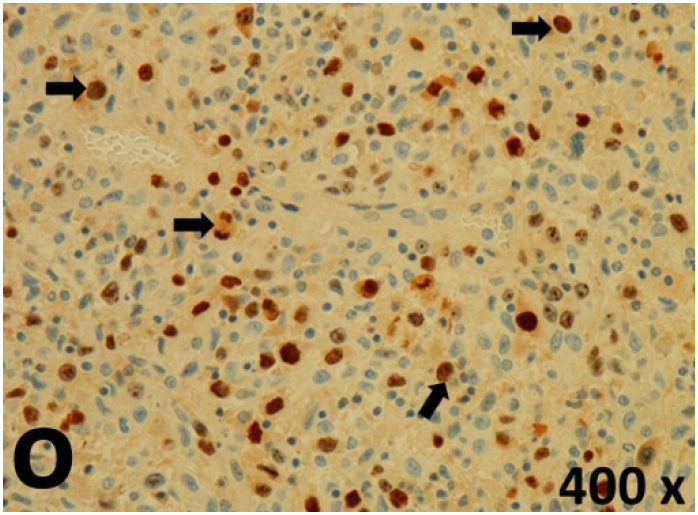	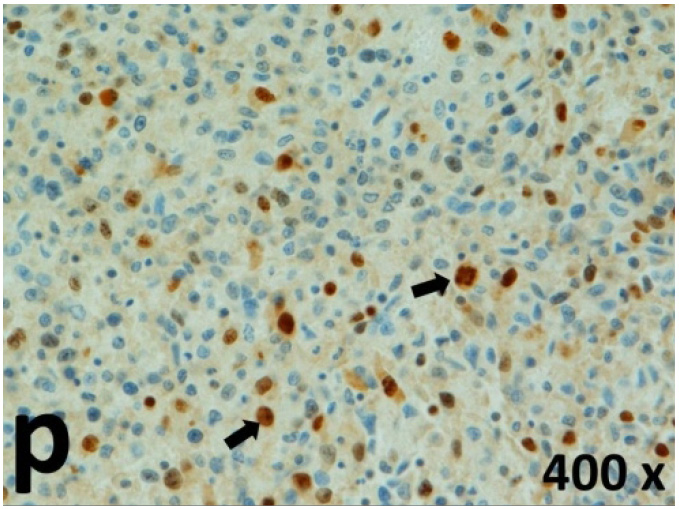
	BICC	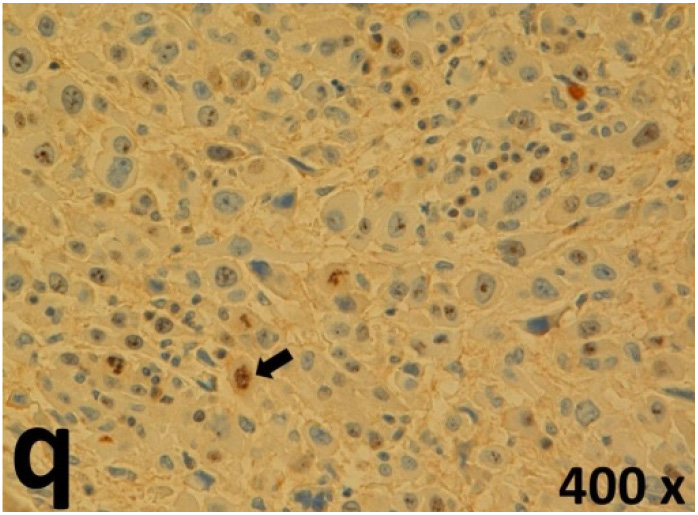	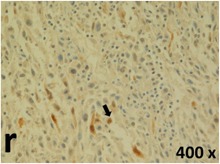

H & E staining revealed central necrosis (black arrows; a–f), GFAP expression (g–l), and Ki-67-positive nuclei (m–r). The black triangles indicate the blood vessels in brain tissues (a–f). The central necrosis area increased rapidly in the control group (a and b) and increased more slowly in the BIC group (c and d), and decreased in the BICC group (e and f). Almost no GFAP expression was observed in the control group (g and h), slight GFAP expression was observed at 4–6 weeks (i), and increased GFAP expression was observed at 8–12 weeks in the BIC group (j). More obvious GFAP expression was observed in the BICC group (k and l). The Ki-67 indices were 22.84%, 45.98%, 15.98%, 11.41%, 7.29%, and 0.79%, as shown in pictures m, n, o, p, q, and r, respectively.

Abbreviations: Gr.: group; H & E: haematoxylin and eosin; GFAP: glial fibrillary acidic protein

## DISCUSSION

Glioblastoma has a poor prognosis and is considered an extremely chemoresistant tumour. Despite modern therapies, malignant gliomas virtually always relapse, typically appearing within 2 cm of the previous resection site. [22, 23] The BBB protects the brain from toxic agents; nevertheless, it also significantly impedes the delivery of therapeutic pharmaceuticals to the brain. Various strategies are used for delivering drugs across the BBB; however, some strategies may structurally harm the barrier. Among the different available approaches, the delivery methods based on nanobiotechnology provide the most desirable prospects to achieve ideal drug delivery [9, 24]. Nanoparticles have their own identity and structural stability according to covalent bonds or strong ionic interaction and may be designed to convey therapeutic agents or other molecules without disturbing the normal brain functions. The potential mechanism of nanoparticle-mediated drug transport across the BBB is determined by the chemistry, architecture, and properties of the nanoparticles [9]. Nanoparticles may be particularly useful for the treatment of disseminated and very aggressive brain tumours [22]. Polymeric nanoparticulates are nano-scaled carriers (1–1000 nm) that are composed of natural or synthetic polymers, where the pharmaceuticals can be embedded in a solid state, dissolved in a solution, or adsorbed or chemically bound to the surface. The employment of polymeric nanoparticles has become one of the most promising approaches for drug delivery to the CNS [25, 26].

The role of chemotherapy in managing GBM has remained controversial because of the frequently disappointing results reported in previous clinical trials. Drug transport may restrict the intracerebral delivery of chemotherapy; however, the major reason of therapeutic failure is mainly tumour resistance to chemotherapy [4]. New chemotherapeutic strategies involve the combined use of multitargeted drugs, cytotoxic chemotherapy and radiotherapy for enhancing therapeutic efficacy and overcoming tumour resistance [6, 27, 28]. Later studies have shown that combining irinotecan and alkylating agents, especially BCNU, increases antitumour influences to a scale that is substantially higher than the additive effects of individual agents [29]. Irinotecan and BCNU act synergistically against CNS tumour cell lines [30]. In a phase II clinical trial, the consolidation of irinotecan with BCNU was active against recurrent or newly identified malignant glioma compared with irinotecan only but with no obvious raised toxicity [17, 30]. Dazzi et al. treated 15 patients with GBM by using the following regimen: BCNU (40 mg/sqm/die) and cisplatin (40 mg/sqm/die) were concurrently managed for 3 days every 3–4 weeks, with radiotherapy of 45-Gy whole cranial irradiation plus a 15-Gy raise on the preoperative volume. This sequential chemoradiotherapy regimen was significantly active in adult patients with newly diagnosed high-grade gliomas [31]. Furthermore, in a phase II study, Grossman et al. reported that the continuous infusion of BCNU and cisplatin followed by cranial irradiation seems to exhibit significant activity and may extend survival in patients with newly diagnosed high-grade astrocytoma [32]. Alba et al. administered a new cisplatin plus bid temozolomide regimen to 50 patients with recurrent GBM. A bid regimen of temozolomide strongly inhibited O^6^-alkylguanine DNA alkyltransferase, and cisplatin reduced the AGAT activity *in vitro*, suggesting a possible synergistic association. Their new cisplatin in combined with bid TMZ regimen was vigorous in chemotherapy-naïve patients with recurrent GBM and caused acceptable toxicity [16].

GBMs are among the most vascular tumours, and hence, tumour-associated vasculature is an attractive therapeutic target [33]. Preclinical models show that antiangiogenic therapy causes temporary vascular normalisation, resulting in increased blood flow. The improved flow subsequently enhances the transport of oxygen and chemotherapeutic agents, thus increasing the efficacy of both radiotherapy and chemotherapy [34, 35]. Furthermore, preventing the development of resistance may require antiangiogenic schemes that give rise to apoptosis or death of the neovasculature. The potential role of circulating endothelial progenitor cells and vascular cooption by tumour cells is also critical [33]. CA4P is a water-dissolvable prodrug of CA4, which is a vascular-targeting and microtubule depolymerising agent. The mechanism underlying the effects of this drug involves the binding of CA4 to the tubulin, resulting in cytoskeletal and then morphological variations in endothelial cells. These alterations raise vascular permeability and disturb the tumour blood flow. Furthermore, in experimental tumours, antivascular influences are observed within few minutes of drug administration and quickly cause widely ischemic necrosis in regions that often show resistance to traditional anticancer treatments [36]. CA4P in combination with bevacizumab appears safe and well tolerated in this dosing schedule. CA4P induces profound vascular changes, which are maintained by bevacizumab [19].

The majority of chemotherapeutic agents should be administered through intravenous infusion, and combined multiple agent therapy potentially raise the systemic toxicity and medical costs. The procarbazine, CCNU (lomustine), and vincristine (PCV) combination regimen has been widely employed to treat malignant glioma. Because of the latent toxicity of BCNU to lung, lomustine has been adopted in the PCV regimen [23, 37]. Due to the high occurrence of hematologic toxicities, the treatment regimen was continuously modified (lomustine, 110 mg/m^2^ on Day 1; procarbazine, 60 mg/m^2^ on Days 8–21; and vincristine, 1.4 mg/m^2^ on Days 8 and 29, with the cycle repeated every 6 weeks) [23]. In the present study, we used biodegradable polymeric nanofibres loaded with three chemotherapeutic agents with different mechanisms and an antiangiogenic agent for achieving sequential and targeted interstitial chemotherapy to avoid systemic toxicity. Surgery remains the fundamental treatment for GBM, through which the tumour bulk is eliminated. The peripheral infiltrating component then becomes the objective of supplementary therapies, such as chemotherapy and radiotherapy [22, 38]. The interstitial chemotherapy proposed in this study was administered through a simple process by placing the drug-loaded membranes onto the brain surface after surgically removing the tumours. Compared with the present interstitial chemotherapy, the Gliadel wafer, the BICC/PLGA nanofibrous membranes can be more easily operated on and cut into any shape to conform to the brain surface after tumour removal. The membrane also provides a longer therapeutic period (more than 8 weeks) than does the Gliadel wafer (5–7 days), which can increase therapeutic efficacy and reduce resistance. The polymeric nanofibres as nanocarriers can protect drugs and deliver them across the BBB for targeting a specific brain cell population [39]. Chemotherapeutic agents and antiangiogenic agents are released from degrading nanofibrous membranes for achieving high and low drug concentrations in the treatment area (brain tissues) and blood (systemic), respectively, thus minimising systemic toxicity. High levels of drug concentrations at the target site and the use of combined multiple chemotherapeutic agents overcome the resistance of tumour cells and enhance the therapeutic efficacy. In our study, the median duration of survival in the BICC, BIC, and control groups was 86.50 ± 48.41, 60.00 ± 44.43, and 22.87 ± 8.21 days, respectively. No statistical significance was observed between the BIC and BICC groups; however, differences between the BICC and control groups (P < 0.001) and between the BIC and control groups (P = 0.01) achieved statistical significance. The survival rate was not statistically significant between the BIC and BICC groups; however, the survival rate of the BIC and BICC groups was statistically higher than that of the control group. Moreover, the measured tumour volumes revealed similar results. The tumour volumes were lower in the BICC group than in the BIC group, but they did not reach statistical significance. The tumour volumes were lower in the BIC and BICC groups than those in the control group (P < 0.001), as determined using the repeated measures mixed model analysis. The results are similar to those of clinical randomised trials. The first-line use of the antiangiogenetic agent bevacizumab showed no improvement of the overall survival in patients with newly diagnosed GBM. Progression-free survival rate was increased but did not reach the expected improvement target [35].

The proliferative index is a potent biological marker for quantitatively estimating the growth of neoplasms, thus aiding in the identification of the prognosis of patients with neoplasms. The Ki-67 labelling index is one of the most effective methods for estimating the proliferative index of the CNS [40, 41]. A case-specific growth period of a GBM was recently suggested; the period was shorter and longer in tumours with high and low cell proliferation indices, respectively [42]. GBM and anaplastic astrocytoma occur from astroglial cells, and GFAP is the most widely used marker of astroglial cells. Malignant astrocytic tumours are GFAP negative, and numerous high-grade gliomas appear to have lower GFAP expression [43, 44]. Similarly, a steady transfection of rat astrocytoma C6 cells with GFAP cDNA suppressed the cells proliferation and extended the cellular processes [45]. In our study, H & E staining revealed that the central necrotic and tumour areas gradually increased and decreased in the BIC and BICC groups, respectively, after the implantation of nanofibrous membranes. The GFAP expression and Ki-67 labelling index demonstrated the most severe malignancy in the control group, followed by the BIC group, and the least severe malignancy in the BICC group.

## MATERIALS AND METHODS

### Preparation of drug-loaded PLGA nanofibrous membrane

Poly(lactid-co-glycolide) polymers, Resomer® RG 503 (lactid:glycolide, 50:50) and RG 756 (lactid:glycolide, 75:25), were commercially obtained from Bochringer Ingelheim, Germany. Cisplatin, bis-chloroethylnitrosourea (BCNU; carmustin), irinotecan, and combretastatin were purchased from Sigma (Steinheim, Germany).

The electrospinning process, an electrostatic fibre fabrication technique that has attracted increasing interest in recent years because of its versatility and potential for applications in diverse fields, was used for producing drug-loaded nanofibrous membranes [46]. The electrospinning setup included a high DC voltage power supply, a syringe pump and needle (internal diameter, 0.42 mm), a ground electrode, and an aluminium sheet. To fabricate the nanofibres, 50:50 poly[(d,l)-lactide-co-glycolide] (PLGA) (240 mg), BCNU (20 mg), irinotecan (20 mg), and cisplatin (20 mg) were first dissolved in 1 mL of 1,1,1,3,3,3-hexafluoro-2-propanol (HFIP; Sigma–Aldrich, USA), whereas 75:25 PLGA (240 mg) and combretastin (60 mg) were dissolved in 1 mL of HFIP. The two solutions were then delivered and sequentially electrospun at room temperature by using a syringe pump at a volumetric flow rate of 1.8 mL/h. The distance between the needle tip and ground electrode and the positive voltage applied to the polymer solutions was 12 cm and 17 kV, respectively. Moreover, the nanoscale fibres were generated by applying a strong electric field on the polymer solution. The electrospun nanofibres were collected in a nonwoven form on the aluminium sheet. The resultant nanofibrous membranes mimic extracellular matrix components more closely than do membranes obtained through conventional techniques. The thickness of the spun bilayered membrane was approximately 0.1 mm.

### Surgical procedures

All animal experiments were performed in accordance with the Taipei Medical University Animal Care and Use Committee guidelines (LAC-2013-0172). A total of 60 adult Wistar rats weighing 200–300 g were included in the *in vivo* drug concentration experiments. All rats were housed in standard facilities with four rats per cage and were provided free access to water and food. The rats were anaesthetised with intraperitoneally injected with 6% chloral hydrate (0.6 mL/kg body weight). Furthermore, the rats were randomly subdivided into nine groups (3 days and 1, 2, 3, 4, 5, 6, 7, and 8 weeks) with five to six rats in each group. After shaving and sterilisation, a 1.5-cm scalp incision was made in the postorbital region. The scalp fascia and muscle were dissected using a scalpel, and craniectomy (approximately 10 × 10 mm) was performed using an electric burr (Figure 1a). After local haemostasis, the biodegradable BCNU, irinotecan, cisplatin, and combretastatin-loaded PLGA (BICC/PLGA) nanofibrous membranes were placed on the surface of the rat brain tissues after craniectomy (Figure 1b). The incision was then closed with 3-0 nylon sutures. Furthermore, after the rats gained consciousness, they were returned to the housing facility. If any intraoperative brain injury or infection (including scalp, skull bone, and brain tissue injury and infection) was observed in the rats, then they were excluded from the study.

### Pharmacokinetics of chemotherapeutic agents

Therats were intraperitoneally administered overdoses of anaesthesia (more than 1.2 mL/kg body weight). Blood samples were collected using syringes through cardiac puncture. Ipsilateral brain tissues (covered with the BICC/PLGA nanofibrous membrane) were also extirpated. Furthermore, wedge-shaped brain tissues (dimensions, 8 × 8 mm and thickness, 8–10 mm) on the brain surface were sliced into five different layers (layers 1–5 from the surface beneath the membrane down to the centre of the brain, with each layer being approximately 1.5-mm thick) by using a rodent brain slicer (Zivic Instruments, USA). Moreover, approximately 0.05 g of brain tissue from each layer was sampled. All specimens (blood and brain tissues of the rats) were collected after 3 days and 1, 2, 3, 4, 5, 6, 7, and 8 weeks. The specimens were centrifuged, and the plasma was collected and stored at −80°C until further analysis. The drug concentrations in the specimens were determined through high-performance liquid chromatography.

### Animal and tumour inoculation

A total of 70 male Wistar rats (age, 4 months; weight, 125–300 g; Charles River, Wilmington, MA, USA) were anaesthetised using an intraperitoneal injection of chloral hydrate (0.6 mL/kg body weight). The anaesthetic effect was monitored using both rail pinch and corneal reflex tests. The animals were secured in a stereotactic frame (Model SAS 64612; ASI Instruments, Warren, MI, USA), a scalp incision in the postorbital region exposed the cranium, and the overlying pericranium was removed using a blunt dissection. Furthermore, craniectomy (approximately 10 × 10 mm) was performed using an electric burr. After adequate haemostasis, a culture medium of 10 μL containing 10^6^ C6 glioma cells was injected into the right temporal region (5 mm below the brain surface) through the implanted catheter at a rate of 1.0 μL/min. After tumour inoculation, the animals were allowed to recover from anaesthesia and were given free access to food and water.

### MRI and microscopic examination

The gross wound appearances were observed daily, and the brain MRI was regularly monitored. All MRI scans were obtained using 7-Tesla Biospec (Bruker, Ettlingen, Germany). Before the membrane implantation, T1- and T2-weighted images were obtained to ensure successful creation of the glioma models, with no epidural, subdural, and intracerebral haemorrhage. All the rats were randomly subdivided into three groups. In the control group, PLGA nanofibrous membranes not embedded with drugs were implanted onto the brain surface of tumour-bearing rats. Furthermore, the BIC/PLGA and BICC/PLGA nanofibrous membranes were implanted into the tumour-bearing rats in the BIC and BICC groups, respectively.

T2-weighted images were obtained as a reference for identifying the tumour region 0, 2, 4, 6, 8, 10, 14, 18, and 22 weeks after the membrane implantation. The tumour volume was reconstructed and calculated using the open-source, FDA-approved Digital Imaging and Communication in Medicine imaging software OsiriX. Any new area of nonenhancing T2 or a fluid-attenuated inversion recovery signal that was consistent with tumour development was considered a progressive disease. The therapy efficacy was evaluated using the tumours' response to the implants as a complete response, partial response, or no response on the basis of the confirmatory MRIs performed after 4 or more weeks. A complete response was defined as no tumour progression (tumour volume always decreased with time); a partial response was defined as a temporary decrease followed by an increase in the tumour volume; and no response was defined as a time-dependent increase in the tumour volume. Furthermore, after a follow-up MRI, 1–2 rats in each group were sacrificed, and their brain tissues were carefully removed for pathological examination (the rats sacrificed for the pathological examinations were excluded from the survival estimation). The brain tissues were fixed in 10% formalin and embedded in paraffin. Coronal sections, 6-μm thick, were prepared and stained with hematoxylin and eosin, and their GFAP expression and the proliferative index Ki-67 were evaluated.

### Statistical analysis

The results are reported as mean ± standard deviation. Statistically significant differences were analysed using the paired sample *t* test by using commercially available Stata software (Version 12.0; Stata, College Station, TX, USA). P < 0.05 was considered statistically significant. Survival data were analysed using the Kaplan–Meier method, with statistical significance determined using the post hoc log-rank test. A repeated measures mixed model was employed for evaluating the effect of different treatments on the implanted tumour growth.

## CONCLUSIONS

In this study, we provided a novel type of interstitial chemotherapy and targeted therapy by using biodegradable drug-loaded nanofibrous membranes. Compared with the only commercially available type of interstitial chemotherapy, the Gliadel wafer, the BICC/PLGA nanofibrous membranes developed in this study provided a longer therapeutic period and a multidrug agent (with different mechanisms of the chemotherapeutic and anti-angiogenic agents) for enhancing therapeutic efficacy and preventing resistance. Moreover, the nanofibres facilitated the penetration of the agents into the BBB and achieved high drug concentrations in the targeted treatment areas (brain tissues). The nanofibrous membranes were not administered through intravenous infusion, thus reducing the systemic toxicity. Furthermore, the experimental results (decreased malignancy, retarded tumour growth, and prolonged survival period in tumour-bearing rats) revealed the efficacy of the BICC/PLGA nanofibrous membranes. Treating GBM by using a single type of therapy may not be effective; therefore, the present study offered a potential treatment regimen for GBM.
